# Proceedings of the Twenty-fourth Session of the Homœopathic Medical Society of Ohio

**Published:** 1889-03

**Authors:** 


Proceedings of the 24th Annual Session of the Homœopathic Medical Society of the State of Ohio. Drs. C. E. Walton and H. Pomeroy, Committee of Publication. 1888.

In this little volume of two hundred odd pages, some interesting and practical questions are discussed. Dr. H. C. Allen gives a partial proving of Magnesia phosphoricum, a remedy which is attracting attention and needs to be further developed.

Dr. R. N. Warren gives "an involuntary proving of Hellebore;" Dr. J. P. Hershberger attempted to report some provings of Cactus, but, as *potencies* had been used in the provings, the I-don't-believe fellows ruled out his report! Yet these potencies *do act.*

